# Editorial: Brain, nutrients, and behavior in orthostatic intolerance

**DOI:** 10.3389/fnhum.2023.1253139

**Published:** 2023-08-03

**Authors:** Kenichi Yamada

**Affiliations:** ^1^Hayakawa Children's Clinic, Niigata, Japan; ^2^Council Niigata for Child Health and Development, Niigata, Japan

**Keywords:** orthostatic intolerance, postural orthostatic tachycardia syndrome (POTS), nutrition, brain and behavior, autoimmune disorders, development

Orthostatic intolerance (OI) is a form of autonomic nervous system (ANS) disturbance characterized by orthostatic dysregulation and a wide variety of ANS symptoms, accompanied by cognitive and psychological issues (Raj et al., 2018). While it typically appears in adolescence, recent reports have shown increasing morbidity as a sequela of COVID-19 (van Campen et al., 2022). Nevertheless, these conditions have not received attention.

Brain and nutritional characteristics associated with cognitive and behavioral alterations in OI need to be elucidated (Blitshteyn, 2022; McWhirter et al., 2022), along with the cardiovascular pathophysiology. Both neuroscientific and nutritional approaches have improved clinical implications and strategies for rational management.

The current Research Topic, titled “*Brain, nutrition, and behavior in orthostatic intolerance”* focuses on the neuroscientific, nutritional, and psychological aspects of OI from both basic and clinical standpoints. It includes eight manuscripts that elucidate their bio-psycho-social properties and add information for a better understanding of their complex pathophysiology.

## COVID-19, inflammation, autoimmunity, and OI

Chadda et al. in a mini review summarized the current worldwide topic of COVID-19 and OI in terms of their shared pathophysiology, which may provide a new window for a better understanding of OI. Given the possible underlying mechanisms, including hypovolemia, neurotropism, inflammation, and autoimmunity, the authors emphasize that future research studies should aim not only to elucidate the underlying mechanisms of dysautonomia but also to educate healthcare professionals to recognize complications and conditions arising from COVID-19, such as postural orthostatic tachycardia syndrome (POTS).

Chan et al. conducted a cohort study of patients who met the pediatric acute-onset neuropsychiatric syndrome (PANS) criteria and compared the clinical characteristics of patients with and without POTS. Nineteen of the 204 patients with PANS showed evidence of POTS, abnormal orthostatic vitals, and persistent POTS symptoms requiring pharmacotherapy for at least 6 months. Moreover, an adjusted logistic regression model showed that a PANS flare was significantly associated with the exacerbation of POTS symptoms. The authors concluded that immune dysfunction, which is present in the PANS, may be implicated in some individuals with PANS and POTS.

Mueller et al. investigated autonomic function testing, which regulates cardiovascular homeostasis and cerebral perfusion in patients with headache, through a review of electronic medical record of 4 years, since acephalgic symptoms, including OI fatigue and cognitive impairment, are common in patients with chronic headache disorders. Sixteen of the 34 patients with chronic headache showed OI, and 11 showed POTS. Reduced cardiovagal reflex sensitivity (BRS-V) predicts chronic headache and POTS. The authors concluded that abnormal autonomic reflexes may play an important role in pain chronification and development of POTS in patients with headaches.

## Nutritional aspects and possible contribution to OI

Nutrition, a potential mediator of immune system maintenance, should be considered as it can be less harmful than neuropsychiatric medicine.

Reed et al. investigated the contribution of the consumption of sugar- and artificially sweetened beverages (SSB and ASB) to cerebral vascular function. The measurement of mean arterial pressure, middle and posterior cerebral artery blood velocities, and end-tidal CO_2_ tension in healthy adults who drank water showed a reduction in the middle cerebral artery (MCA) compared to those who drank water, whereas cerebrovascular reactivity to CO_2_ in posterior cerebral artery (PCA CVRco_2_) did not differ between beverages. These findings provide a better understanding of the worsening of OI symptoms by drinking SSB or ASB, as several other studies have reported.

Worley et al. demonstrated the potential role of beetroot juice (BRJ) in cerebrovascular function and cardiovagal baroreflex to improve peripheral endothelial function and vascular compliance, likely due to increased nitric oxide bioavailability using oscillatory and static lower-body genitive pressures with CO_2_ inhalation under BRJ consumption. The results indicated that cerebral autoregulation, measured using middle cerebral artery blood velocity (transcranial Doppler) and indexed through coherence, was largely unaffected by an acute BRJ bolus. Based on these preliminary findings in healthy individuals, investigation of individuals with OI is needed to explore a new nutritional approach.

Roger et al. demonstrated that brain function could be altered through malnutrition by evoked-related potentials (ERPs). The authors found that previously malnourished adults had a higher rate of omission errors on the task relative to controls, and that ERP was significantly different in participants with a history of early malnutrition. The authors concluded that these findings may be linked to attentional and executive function deficits that have been previously reported in childhood, as they are typically associated with impaired conflict monitoring and/or attention deficits.

## Other variables potentially contributing to behavior in OI

Zhang and Zhang described the structural and functional characteristics of the brains of individuals living at high altitudes (HAs). Specific brain regions, including the insular cortex, cingulate cortex, cerebellum, and hippocampus, were affected by HA exposure. These regions show altered gray matter volumes and neuronal activity, as detected via electrophysiological recordings and functional magnetic resonance imaging. Given the possible association of the pathophysiology of OI and gravitational and air pressure forces, which may be modulated by HA, it may provide a new clue to manage OI in terms of HA-like environmental settings and adaptation.

Alhamdan et al. investigated multisensory processing and age-related improvements, which could reflect higher-level perceptual and cognitive abilities in children with OI that are likely to be reduced. The Bayesian results provided decisive evidence for age-group differences in multisensory motor reactions and visuomotor processing tasks. The authors concluded that multisensory integration is likely to continue into late childhood and early adolescence, indicating that these observations can be used to assess alterations in the brain and behavior during OI.

## Conclusive remark

Taken together, the eight studies on this topic aimed to provide an overview of the possibilities for new or alternative viewpoints on OI. We hope that the current edition will facilitate a next step toward better understanding and management of individuals with OI.

## Author contributions

The author confirms being the sole contributor of this work and has approved it for publication.
